# P-1934. Differences between the Reporting of Post-COVID-19 Symptoms and the Associated Health-Related Quality of Life during and after COVID-19 Infection - results of the Cross-Sectoral Platform of the German National Pandemic Cohort Network

**DOI:** 10.1093/ofid/ofae631.2093

**Published:** 2025-01-29

**Authors:** Sina M Hopff, Katharina Appel, Isabel Bröhl, Carolin Nürnberger, Patricia Wagner, Margarete Scherer, Christine Allwang, Sabine Blaschke, Hiwa Dashti, Clara de Angelis, Eckard Hamelmann, Axel Hamprecht, Stefan Hansch, Frank Hanses, Peter Heuschmann, Thomas Illig, Achim J Kaasch, Nadja Käding, Robin Kobbe, Dagmar Krefting, Kristin Lehnert, Christina Lemhöfer, Thomas Lücke, Susana M Nunes de Miranda, Shimita Raquib, Jens-Peter Reese, Christoph Römmele, Julia Schmidt, Petra Schulze, Johannes J Tebbe, Maria J GT Vehreschild, Christoph Wyen, Janne J Vehreschild

**Affiliations:** University of Cologne, Faculty of Medicine and University Hospital Cologne, Department I of Internal Medicine, Center for Integrated Oncology Aachen Bonn Cologne Duesseldorf, Cologne, Nordrhein-Westfalen, Germany; Goethe University Frankfurt, Faculty of Medicine, Institute for Digital Medicine and Clinical Data Science, Germany, Frankfurt, Hessen, Germany; University of Cologne, Faculty of Medicine and University Hospital Cologne, Department I of Internal Medicine, Cologne, Germany, Cologne, Nordrhein-Westfalen, Germany; University of Wuerzburg, Faculty of Medicine, Institute for Clinical Epidemiology and Biometry, Wuerzburg, Germany; University Hospital Wuerzburg, Institute for Medical Data Sciences, Wuerzburg, Germany, Würzburg, Nordrhein-Westfalen, Germany; University of Cologne, Faculty of Medicine and University Hospital Cologne, Department I of Internal Medicine, Cologne, Germany, Cologne, Nordrhein-Westfalen, Germany; Goethe University Frankfurt, Faculty of Medicine, Institute for Digital Medicine and Clinical Data Science, Germany, Frankfurt, Hessen, Germany; TUM School of Medicine and Health – Clinical Department of Psychosomatic Medicine and Psychotherapy, University Medical Center, Technical University of Munich, Munich, Bayern, Germany; University Medical Center Goettingen, Emergency Department, Göttingen, Niedersachsen, Germany; Practice for General Medicine Dashti, Eberswalde, Germany, Eberswalde, Brandenburg, Germany; Department of Gastroenterology, Hepatology and Infectious Diseases, Medical Faculty and University Hospital Düsseldorf, Heinrich-Heine-University Düsseldorf, Moorenstraße 5, 40225, Düsseldorf, Germany, Düsseldorf, Nordrhein-Westfalen, Germany; Bielefeld University, Medical School and University Medical Center OWL, Protestant Hospital of the Bethel Foundation, Department of Pediatrics, Bielefeld, Nordrhein-Westfalen, Germany; Carl von Ossietzky University Oldenburg and Klinikum Oldenburg, Institute of Medical Microbiology and Virology, Oldenburg, Niedersachsen, Germany; Department for Infection Control and Infectious Diseases, University Hospital Regensburg, Regensburg, Bayern, Germany; Emergency Department and Department for Infection Control and Infectious Diseases, University Hospital Regensburg, Regensburg, Germany, Regensburg, Bayern, Germany; University of Wuerzburg, Faculty of Medicine, Institute for Clinical Epidemiology and Biometry, Wuerzburg, Germany; University Hospital Wuerzburg, Institute for Medical Data Sciences, Wuerzburg, Germany, Würzburg, Nordrhein-Westfalen, Germany; Hannover Medical School, Hannover Unified Biobank, Hannover, Germany, Hannover, Niedersachsen, Germany; Institute of Medical Microbiology and Hospital Hygiene, University Hospital Magdeburg, Medical Faculty of Otto von Guericke University Magdeburg, Germany, Magdeburg, Sachsen-Anhalt, Germany; Department of Infectious Diseases and Microbiology, University-Hospital Schleswig-Holstein Campus/Lübeck, Lübeck, Germany, Lübeck, Schleswig-Holstein, Germany; Institute for Infection Research and Vaccine Development (IIRVD), University Medical Centre Hamburg-Eppendorf, Hamburg, Germany; Department of Infectious Disease Epidemiology, Bernhard Nocht Institute for Tropical Medicine, Hamburg, Germany, Hamburg, Hamburg, Germany; Department of Medical Informatics, University Medical Center Göttingen, Göttingen, Germany; Campus Institute Data Sciences, Göttingen, Germany, Göttingen, Niedersachsen, Germany; DZHK (German Center for Cardiovascular Research), University Medicine Greifswald, 17475 Greifswald, Germany; Department of Internal Medicine B, University Medicine Greifswald, 17475 Greifswald, Germany, Greifswald, Mecklenburg-Vorpommern, Germany; Institute of Physical and Rehabilitation Medicine, Jena University Hospital/Friedrich-Schiller-University Jena, Jena, Germany, Jena, Thuringen, Germany; Ruhr-University Bochum, St. Josef-Hospital, University Hospital of Pediatrics and Adolescent Medicine, Bochum, Nordrhein-Westfalen, Germany; University of Cologne, Faculty of Medicine and University Hospital Cologne, Department I of Internal Medicine, Center for Integrated Oncology Aachen Bonn Cologne Duesseldorf, Cologne, Nordrhein-Westfalen, Germany; Goethe University Frankfurt, Faculty of Medicine, Institute for Digital Medicine and Clinical Data Science, Germany, Frankfurt, Hessen, Germany; Insitute for Clinical Epidemiology and Biometry, Julius Maximilians Universität Würzburg, Würzburg, Bayern, Germany; Clinic for Internal Medicine III - Gastroenterology and Infectious Diseases, University Hospital of Augsburg, Augsburg, Germany, Augsburg, Bayern, Germany; Insitute for Clinical Epidemiology and Biometry, Julius Maximilians Universität Würzburg, Würzburg, Bayern, Germany; University Hospital Würzburg, Department of Internal Medicine II, Division of Infectious Diseases, Oberdürrbacherstraße 6, Würzburg, Germany, Würzburg, Bayern, Germany; University Medical Center East Westphalia-Lippe, Klinikum Lippe, Department of Gastroenterology and Infectious Disease, Lippe, Nordrhein-Westfalen, Germany; Department of Internal Medicine, Infectious Diseases, University Hospital Frankfurt Goethe University Frankfurt, Frankfurt am Main, Hessen, Germany; Medical practice Ebertplatz, Cologne, Germany, Cologne, Nordrhein-Westfalen, Germany; Goethe University Frankfurt, Faculty of Medicine, Institute for Digital Medicine and Clinical Data Science, Germany, Frankfurt, Hessen, Germany

## Abstract

**Background:**

Post-COVID-19 syndrome (PCS) prevalences range from 10% up to 45%, caused by heterogeneous definitions, different study designs and survey methods, and sometimes misattribution of unrelated comorbidities to PCS. First correlations between PCS and health-related quality of life (HrQoL) are described. This study analyzes the course and differences between PCS-related symptoms and the associated HrQoL in a large multicenter cohort.

**Table 1:**
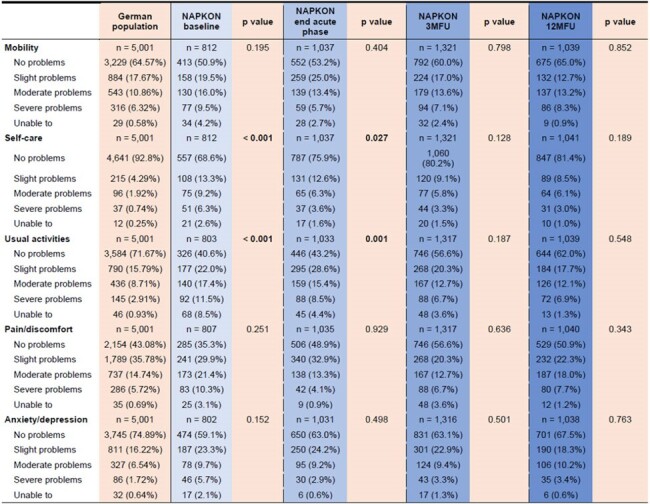
Development of Health-related Quality of Life (HrQoL) in COVID-19 patients compared to the healthy population

Comparisons of the frequency distribution of the individual EQ-5D-5L dimensions of the NAPKON cohort at the time of COVID-19 diagnosis (baseline), end of the acute phase, as well as the 3- (3MFU) and 12-month follow-up (12MFU) with those of a general German population according to Grochtdreis et. al (DOI: 10.1007/s10198-019-01054-1). Significance levels were computed using Person’s chi square test with p < 0.05 = significant.

**Methods:**

Prospectively collected data from COVID-19 patients enrolled in the Cross-Sectoral Platform of the German National Pandemic Cohort Network (NAPKON) were used to describe HrQoL by EQ-5D-5L. Interviews were conducted at the time of COVID-19 diagnosis, the end of acute phase, and during the 3- (3MFU) and 12-month follow-ups (12MFU). Results were compared with pre-pandemic German population data. Group comparisons were assessed using Pearson’s chi square test. EQ-5D-5L index scores were stratified by the severity of PCS according to a PCS score (DOI: 10.1016/j.eclinm.2022.101549) and correlations were assessed by Pearson’s correlation coefficient (p < 0.05 significant, respectively).

**Figure 1:**
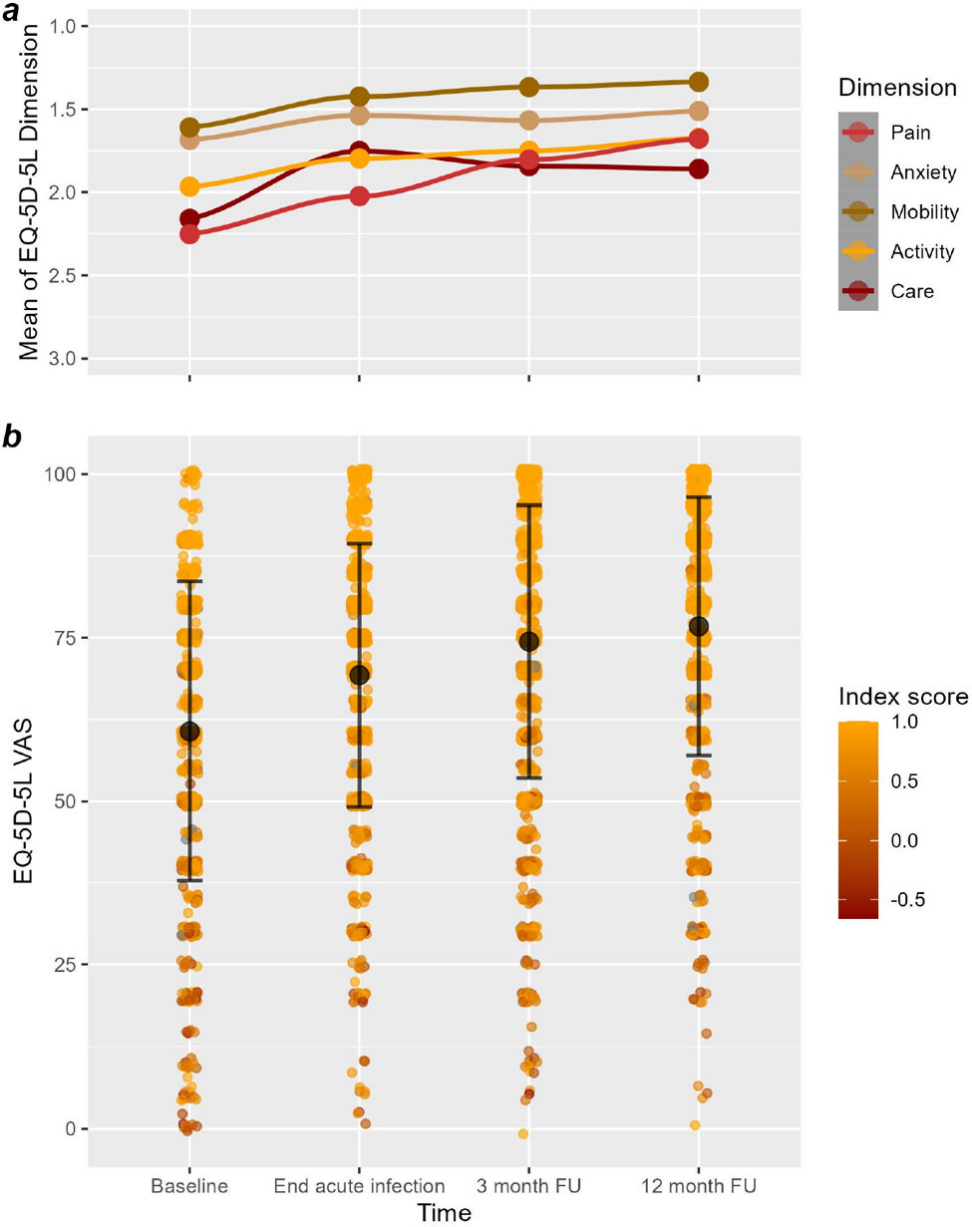
Development of EQ-5D-5L expressions over the observation period (a) The development of means of the 5 EQ-5D-5L dimensions pain, anxiety, mobility, usual activities (activity), and self-care (care) of COVID-19 patients of the NAPKON cohort are demonstrated over the observation time (1 = no problems, 2 = slight problems, 3 = moderate problems, 4 = severe problems, 5 = unable to). (b) EQ-5D-5L Visual Analogue Scale (VAS) values are indicated at the time of COVID-19 diagnosis (baseline), end of the acute phase, as well as the 3- and 12-month follow-up (FU). In addition, the level of the EQ-5D-5L index score is visualized by a color scale.

**Results:**

We analyzed 1,494 patients from 39 study sites (median age 55, range 18-94; 42% female). During the acute phase, COVID-19 patients reported more difficulties in self-care (p < 0.001) and usual activities (p < 0.001) than the healthy population. HrQoL of COVID-19 patients constantly improved towards the 3MFU and 12MFU hence there was no longer a difference compared to the general population (Table 1). The courses of individual dimensions were heterogeneous (Figure 1). However, at the 3MFU 61% and at the 12MFU 57% of the patients still had PCS-related symptoms with a significant correlation between the EQ-5D-5L index score and the reported severity of PCS (3MFU: r = -0.39, p < 0.001; 12MFU: r = -0.48, p < 0.001).

**Conclusion:**

We found no significant impairment in HrQoL up to 12 months after COVID-19 diagnosis compared to the healthy population, despite continued patient reports on PCS-related symptoms. While severity of symptoms was correlated with HrQoL, the overall impact of PCS was lower than expected. We conclude that incorporating HrQoL assessments alongside symptom-based questionnaires is crucial for accurately measuring PCS impact on daily life.

**Disclosures:**

Sina M. Hopff, MD, Tillotts: Travel costs Christine Allwang, Dr. med., Ärzte- und Ärztinnenverband Long COVID: Board Member|BMBF German Federal Ministry of Education and Research: Grant/Research Support Sabine Blaschke, Prof. Dr. med., BMBF NAPKON NUM 2.0 Grant No. 01KX2121: Grant/Research Support Stefan Hansch, n/a, ETFS and equities (not specific to the medical sector): Stocks/Bonds (Private Company)|Gilead: Travel grants; Attending workshop (training) Frank Hanses, n/a, Akademie für Infektionsmedizin: Honoraria|Bayr. Landesärztekammer: Honoraria|Bayr. Staatsministerium für Gesundheit und Pflege: Grant/Research Support|Correvio / Advanz: Honoraria|DGINA: Honoraria|Sobi, GSK: Board Member Peter Heuschmann, Prof. Dr. med., Bavarian State: Grant/Research Support|Charité – Universitätsmedizin Berlin (within Mondafis; supported by an unrestricted research grant to the Charité from Bayer): Grant/Research Support|European Union: Grant/Research Support|Federal Joint Committee (G-BA) within the Innovationfond: Grant/Research Support|German Cancer Aid: Grant/Research Support|German Heart Foundation: Grant/Research Support|German Ministry of Research and Education (within the NUM): Grant/Research Support|German Parkinson Society: Grant/Research Support|German Research Foundation: Grant/Research Support|Participation on DSMB in publicly funded studies (by German Research Foundation, German Ministry of Research, Foundations): Board Member|University Göttingen (within FIND-AF randomized; supported by an unrestricted research grant to the University Göttingen from Boehringer-Ingelheim): Grant/Research Support|University Hospital Heidelberg (within RASUNOA-prime; supported by an unrestricted research grant to the University Hospital Heidelberg from Bayer, BM: Grant/Research Support|University Hospital Würzburg: Grant/Research Support Achim J. Kaasch, Prof. Dr. med., Network of University Medicine (NUM), funded by the German Federal Ministry of Education and Research (BMBF) (FKZ: 01KX2121): Grant/Research Support Dagmar Krefting, Prof. Dr., BMBF (Federal Ministry of Education and Research ): Grant/Research Support Christina Lemhöfer, Dr. med., Federal Joint Committee (G-BA) within the Innovationfond: Grant/Research Support|German Society of Physical and Rehabilitation Medicine (Deutsche Gesellschaft für Physikalische und Rehabilitative Medizin e.V. (DGPRM)): Board Member|Network University Medicine (NUM)/ German Federal Ministry of Education and Research (BMBF): Speaker Physical and Rehabilitation Group|Physikalische Medizin, Rehabilitationsmedizin, Kurortmedizin’: Board Member Jens-Peter Reese, Univ.-Prof. Dr. med., Bavarian State (ministry for science and the arts), DigiOnko: Grant/Research Support|Federal Joint Committee (G-BA) within the Innovationfond, Peri-OP, StaerkeR: Grant/Research Support|German Center for Lung Research, within PASSION: Grant/Research Support|German Ministry of Health (BMG): Expert Testimony|German Ministry of Research and Education: Grant/Research Support|Landesaerztekammer Hessen: Honoraria Julia Schmidt, n/a, Contract of employment at the University of Wuerzburg: Grant/Research Support Maria JGT Vehreschild, Prof. Dr. med., 3M: Honoraria|Ärztekammer Niedersachsen: Honoraria|ADKA: Honoraria|Akademie für Infektionsmedizin: Honoraria|Astellas: Honoraria|Berliner Dialy Seminar: Honoraria|Biontech: Grant/Research Support|CED Service: Honoraria|DiaLog Service: Honoraria|EUMEDICA: Advisor/Consultant|EUMEDICA: Honoraria|Falk Foundation: Honoraria|Ferring: Honoraria|Forum für medizinische Fortbildung Gmbh: Honoraria|GILEAD: Honoraria|Heel: Grant/Research Support|Helios Kliniken: Honoraria|Institute Merieux: Honoraria|Janssen: Honoraria|Kit Kongress: Honoraria|Klinikum Essen: Honoraria|Klinikum Leverkusen: Honoraria|Lahn-Dill-Kliniken: Honoraria|Landesärztekammer Hessen: Honoraria|Limbach Gruppe SE: Honoraria|MaaT: Advisor/Consultant|MSD: Advisor/Consultant|MSD: Grant/Research Support|MSD: Honoraria|Pfizer: Honoraria|Roche: Advisor/Consultant|Roche: Grant/Research Support|SD Biosensor: Grant/Research Support|St. Johannes Hospital: Honoraria|St. Josef Hospital: Honoraria|SUMIT OXFORD Ltd.: Honoraria|Tillotts: Advisor/Consultant|Tillotts: Honoraria|Uniklinik Frankfurt: Honoraria|Uniklinik Köln: Honoraria|Uniklinik Karlsruhe: Honoraria|Universitätsklinikum Freiburg: Honoraria|Universitätsklinikum Heidelberg: Honoraria Janne J. Vehreschild, Prof. Dr. med., Ärztekammer Hessen: Honoraria|Ärztekammer Nordrhein: Honoraria|Academy for Infectious Medicine: Board Member|Academy for Infectious Medicine: Honoraria|APOGEPHA: Honoraria|Astellas Pharma: Board Member|Astellas Pharma: Grant/Research Support|Astellas Pharma: Honoraria|Back Bay Strategies: Honoraria|Basilea: Board Member|Basilea: Grant/Research Support|Basilea: Honoraria|Biontech: Board Member|Biontech: Honoraria|Deutsches Zentrum für Luft- und Raumfahrt (DLR): Grant/Research Support|European Union: Grant/Research Support|German Cancer Consortium (DKTK): Grant/Research Support|German Cancer Consortium (DKTK): Honoraria|German Cancer Consortium (DKTK): Support for attending meetings|German Centre for Infection Research (DZIF): Board Member|German Centre for Infection Research (DZIF): Grant/Research Support|German Centre for Infection Research (DZIF): Honoraria|German Centre for Infection Research (DZIF): Support for attending meetings|German Federal Ministry of Education and Research (BMBF): Grant/Research Support|German Federal Ministry of Health (BMG): Grant/Research Support|German Network University Medicine: Board Member|German Network University Medicine: Grant/Research Support|German Network University Medicine: Honoraria|German Network University Medicine: Support for attending meetings|German Society for Infectious Diseases (DGI): Board Member|German Society for Infectious Diseases (DGI): Honoraria|German Society for Infectious Diseases (DGI): Support for attending meetings|German Society for Internal Medicine (DGIM): Board Member|German Society for Internal Medicine (DGIM): Honoraria|German Society for Internal Medicine (DGIM): Support for attending meetings|Gilead: Board Member|Gilead: Grant/Research Support|Gilead: Honoraria|Janssen: Board Member|Janssen: Honoraria|Merck/MSD: Board Member|Merck/MSD: Grant/Research Support|Merck/MSD: Honoraria|Molecular Health: Honoraria|NordForsk: Honoraria|Pfizer: Board Member|Pfizer: Grant/Research Support|Pfizer: Honoraria|Rigshospitalet Copenhagen: Grant/Research Support|Shionogi: Honoraria|University Hospital Aachen: Honoraria|University Hospital Aachen: Support for attending meetings|University Hospital Freiburg/ Congress and Communication: Honoraria|University Hospital Oldenburg: Honoraria|University Manchester: Board Member|University Manchester: Honoraria|University Manchester: Support for attending meetings|University of Bristol: Grant/Research Support

